# Nanofibers for drug delivery – incorporation and release of model molecules, influence of molecular weight and polymer structure

**DOI:** 10.3762/bjnano.6.198

**Published:** 2015-09-25

**Authors:** Jakub Hrib, Jakub Sirc, Radka Hobzova, Zuzana Hampejsova, Zuzana Bosakova, Marcela Munzarova, Jiri Michalek

**Affiliations:** 1 The Institute of Macromolecular Chemistry, Academy of Sciences of the Czech Republic, Heyrovsky Sq. 2, 162 06 Prague 6, Czech Republic; 2 Department of Analytical Chemistry, Faculty of Science, Charles University in Prague, Albertov 2030, 128 43 Prague 2, Czech Republic; 3 Nanovia Ltd., Podkrusnohorska 271, 436 03 Litvinov-Chuderin, Czech Republic

**Keywords:** nanofibers, nanofibrous carriers, needle-free electrospinning, release kinetics

## Abstract

Nanofibers were prepared from polycaprolactone, polylactide and polyvinyl alcohol using Nanospider^TM^ technology. Polyethylene glycols with molecular weights of 2 000, 6 000, 10 000 and 20 000 g/mol, which can be used to moderate the release profile of incorporated pharmacologically active compounds, served as model molecules. They were terminated by aromatic isocyanate and incorporated into the nanofibers. The release of these molecules into an aqueous environment was investigated. The influences of the molecular length and chemical composition of the nanofibers on the release rate and the amount of released polyethylene glycols were evaluated. Longer molecules released faster, as evidenced by a significantly higher amount of released molecules after 72 hours. However, the influence of the chemical composition of nanofibers was even more distinct – the highest amount of polyethylene glycol molecules released from polyvinyl alcohol nanofibers, the lowest amount from polylactide nanofibers.

## Introduction

To date, numerous drug delivery systems have been developed, such as hydrogels that carry drugs or highly sophisticated electronic microchips [1–2]. The required release rates of the therapeutic agents depend on the medicinal application; for example, the optimal release time of hormones is in the range of months, while peroral administration requires that the drug is released as fast as possible [3]. Nano-shaped materials are advantageous for the rapid release of the drugs due to their high surface area/volume ratio.

Due to the internal architecture, nanofibers are well suited for various medicinal applications, such as carriers for cell cultivation [4–5], tissue engineering scaffolds [6] or wound dressings [7]. The incorporation of biologically or pharmacologically active compounds into the nanofibers may be very useful for these applications [8].

Although several methods of nanofiber preparation have been invented [9], electrospinning technique can be considered as a simple and versatile method for the production of continuous polymeric nanofibrous mats formed of nano- to micro-sized fibers [10–14]. Moreover, this fabrication enables to set-up process parameters for facile control of nanofibrous mat properties such as surface area, fiber diameter, porosity, and thickness [15].

In the recent years, much effort has been devoted to modifying the electrospinning process, so coaxial, multi-jet, or side-by-side techniques were developed [16–17]. However, these techniques are of a little interest in terms of potential mass production.

In contrast, Nanospider^TM^ technology as an alternative approach based on a needle-free method represents the perspectives for industry due to the high production capacity, stability and easy maintenance [18–19]. This technology is relatively universal and nanofibrous materials from a variety of polymers can be obtained. Moreover, adjusting the process parameters such as the concentration of polymer in solution, electric field strength, tip-to-collector distance or temperature the materials enables to control the final structure of the prepared materials [6,20].

Generally, nanofibers that carry drugs follow several basic designs – nanofibers with homogenous structures in which the drug is dispersed throughout the polymer matrix, core–shell nanofibers for which the matrix carrying the drug is covered by pure polymer [21–22] and nanofibers with the pharmacologically active compounds immobilized on their surface [16,23]. Nowadays, also more sophisticated structures of nanofibrous mats are described, such as multilayer constructs [16]. The two basic fiber designs are the primary factors that affect the diffusion mechanism and drug release. For homogenous nanofibers, the rate of release decreases with time, because the drug must travel progressively longer distances to diffuse to the fiber periphery, which requires more time. Contrary, the core–shell design provides the delivery system with the diffusion rate of the therapeutic agent stable throughout the life. The structure of the nanofibrous drug delivery system plays a key role in the drug release process. The fiber diameter, specific surface area, size and total volume of pores significantly influence the convection and diffusion of the liquid in which the nanofibers are immersed. Therefore, the drug release is also influenced. The great advantage of nanofibrous materials is that their structure, i.e., their fiber diameter, density and thickness of the nanofibrous layer, can be tailored to various requirements by varying the process parameters [24].

In this work, we designed a nanofibrous carrier in which the model molecule is dispersed throughout the polymer matrix. For the purposes to evaluate the influence of molecular size on the release rate and the total released amount in general, polyethylene glycols with molecular weights of 2,000, 6,000, 10,000 and 20,000 g/mol were selected as model molecules. Polyethylene glycol (PEG) is a hydrophilic polyether commercially available in various molecular weights with narrow distribution. It is widely used in various medical applications, for example as surfactant, solvent or tablet excipient. Apart from these applications the PEG has significant effect on the drug release. It has been shown that addition of PEG molecules is an efficient way to modify the release of hydrophobic paclitaxel from poly(lactic acid-*co*-glycolic acid) matrix [25] or proteins from lipidic implants [26]. In present work the PEGs were added to the solutions of polymers and were incorporated in nanofibers made from polycaprolactone (PCL), polylactide (PLA) and polyvinyl alcohol (PVA) during electrospinning. The release behavior of these molecules into the water environment was investigated and the results are discussed in terms of molecular weight of PEGs and chemical composition of nanofibers.

## Results and Discussion

**Figure 1:**
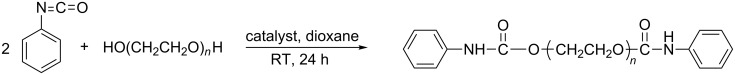
Derivatization reaction scheme of PEG with phenyl isocyanate.

Prior to use, linear PEGs were terminated with phenyl isocyanate to incorporate spectrophotometrically detectable groups (Figure 1). The reaction products were confirmed by NMR, elemental analysis, IR spectroscopy and melting point measurements.

**Figure 2:**
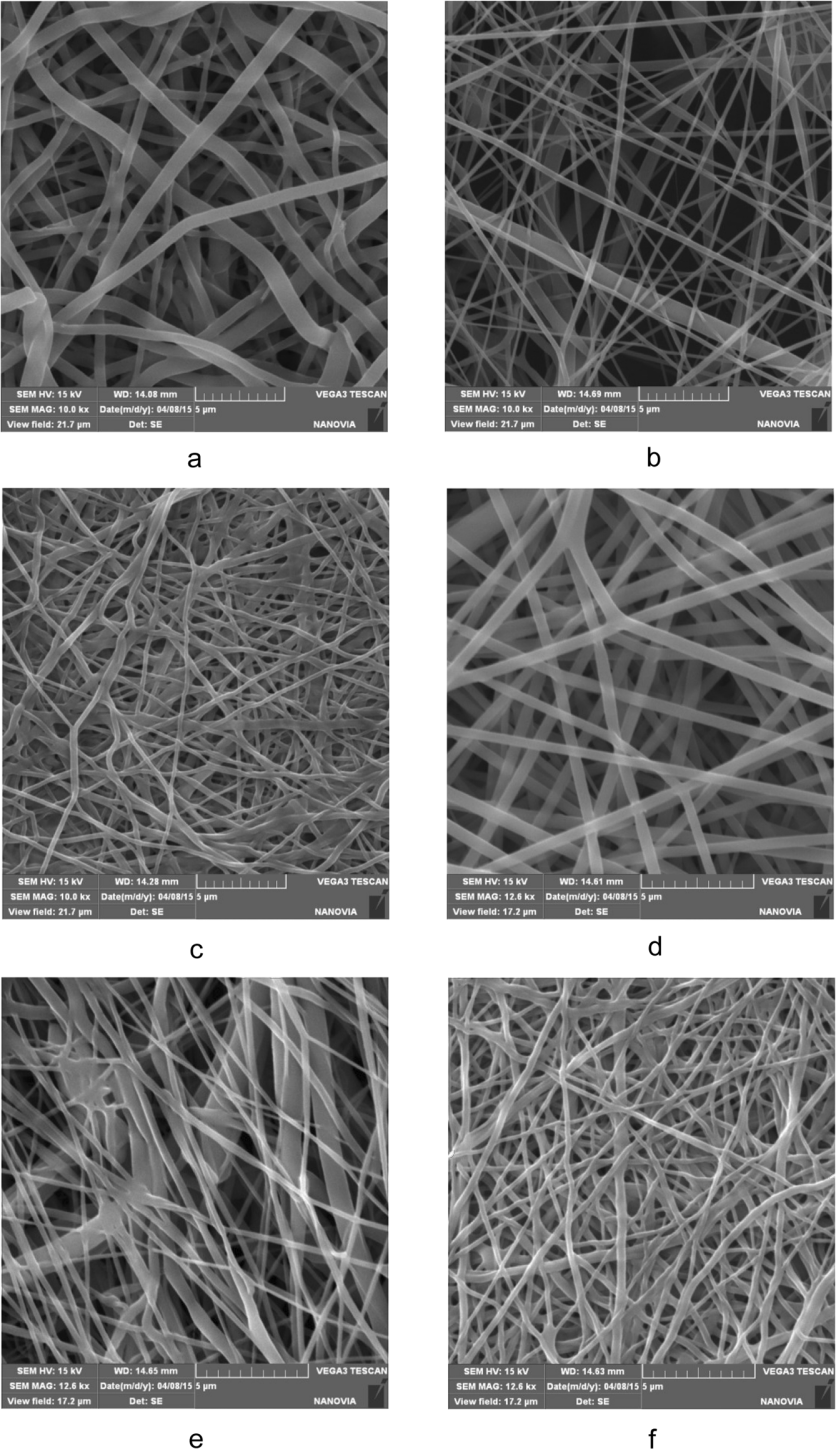
SEM images of PLA (a, d), PCL (b, e) and PVA (c, f) nanofibers prepared without (a–c) and with (d–f) addition of PEG 20 molecules.

The needle-free electrospinning process was optimized for each type of nanofiber with respect to the different physicochemical properties of polymers. SEM images revealed that the textures of all resultant samples were homogenous and free of heterogeneities or artifacts (Figure 2a–c). It can be expected that the addition of PEGs will change the physicochemical properties of the polymer solution in electrospinning, however, it was not noticeable in concentration of PEG 3%. The addition of PEG molecules to the electrospun mixture did not noticeably influence the nanofiber structure. According to the results of morphological characterization, the nanofibrous structures remained similar to those of nanofibers without model molecules, even for nanofibers that contained PEG 20 (Figure 2d–f). Clusters or other artifacts were not detected.

The properties and behavior of each polymer during electrospinning were unique. The process parameters, such as the polymer mixture composition, voltage, electrode distance, temperature or humidity, were individually and precisely adjusted in order to produce structures that were as similar as possible. However, the morphological characterization revealed several differences in the parameters of resultant samples (Table 1). The thinnest fibers with a mean fiber diameter 157 nm were prepared from PVA, and the thickness of PCL nanofibers (179 nm) was almost similar to this value. PLA fibers were the thickest, with a diameter of 282 nm. The surface areas corresponded to the fiber diameters; the surface area was largest for the thinnest PVA fibers (7.7 m^2^/g) and smallest for PLA (4.7 m^2^/g). These differences are due to the needle-free electrospinning method. Needle-free electrospinning does not allow the fine-tuning of parameters, but the disadvantage of this method is balanced by the fact that it may allow the large-scale production. The possible effect of these characteristics is discussed further below.

**Table 1:** Morphological characterization of nanofibrous carriers.

	fiber diameter (nm)	porosity (%)	surface area (m^2^/g)

PCL	179	77.4	6.0
PVA	157	78.2	7.7
PLA	282	86.6	4.7

The porosities determined by mercury porosimetry ranged from 77.4% for PCL nanofibers to 86.6% for PLA nanofibers. These differences may have slightly influenced the release of incorporated molecules. However, the mercury porosimetry measurements were conducted in a vacuum. In an aqueous environment, in which the nanofibers are supposed to be used, the porosity may differ due to the variable water content between nanofibers, which is the result of their variable swelling.

**Figure 3:**
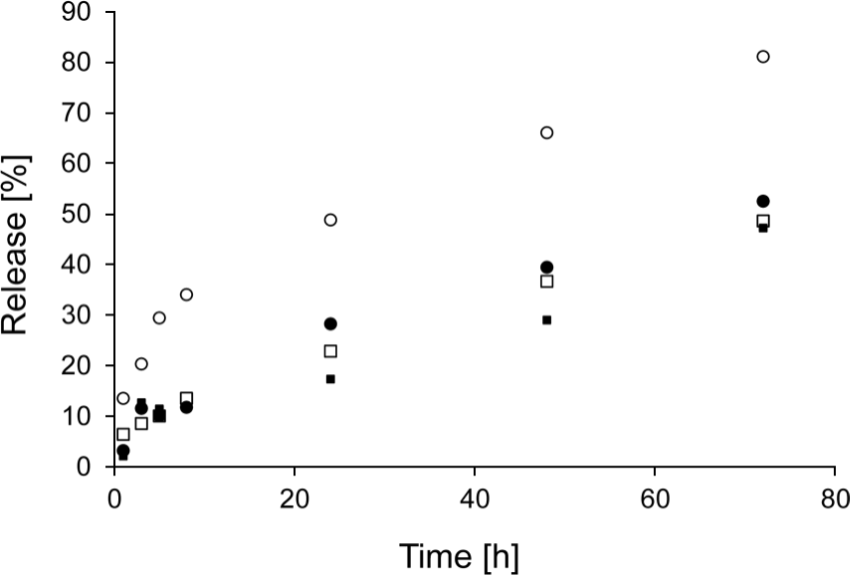
Release of PEG from PVA nanofibers, PEG 2 (filled squares), PEG 6 (open squares), PEG 10 (filled circles), PEG 20 (open circles).

**Figure 4:**
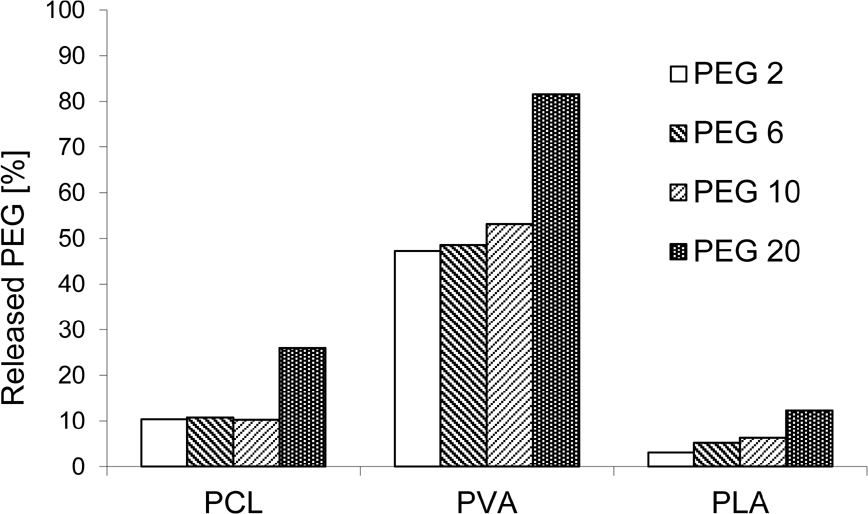
Amount of PEG molecules of various molecular weights released from PCL, PVA and PLA nanofibers immersed to distilled water within 72 h at 20 °C.

The release experiments revealed two trends – the release rate depends on the molecular weight of model PEGs as well as on the type of nanofibrous carrier. Although we expected that long molecules will be anchored in nanofibrous structures and so more strongly retained, we observed the opposite effect. Larger molecules were apparently released faster than smaller ones (see Figure 3). Figure 4 depicts the amount of PEG molecules released within 72 hours. Except for the amount of PEG 10 released from PCL nanofibers, which we attribute to random error, the percentage of PEG that was released positively correlated with molecular size. The most distinct increase in the release rate was observed for PEG 20, whose release was approximately 30% higher than that of PEG 10. This effect was attributed to the insufficient interweaving of PEG and chains of the polymer matrix during the electrospinning process. Expecting the well interweaving of PEG molecules, their release is primarily influenced by the dissolution rate and consequent transport through the material, i.e., the molecules with lower molecular weight and so higher mobility should release faster. However, our samples behaved differently. Our findings suggest that chains of PEG likely form domains that are separated from nanofibers and these domains accelerate their release from the material. This effect is more apparent in longer chains. Shorter chains are more mobile, which allows them to partially penetrate the nanofibers. In this case, the distance from the surface controls the dissolution rate. The longer chains of PEG 20 contain parts of molecules which remain near the surface and are more accessible to water. This effect accelerates the release of longer PEG chains compared to shorter ones.

The formation of PEG domains can be also supported by the formation of a crystalline phase. Larger PEGs are more likely to crystalize. During the formation of a crystalline phase, the domains of the crystalline polymer are formed separately from the remaining system, which makes them more accessible to water and easier to release.

**Figure 5:**
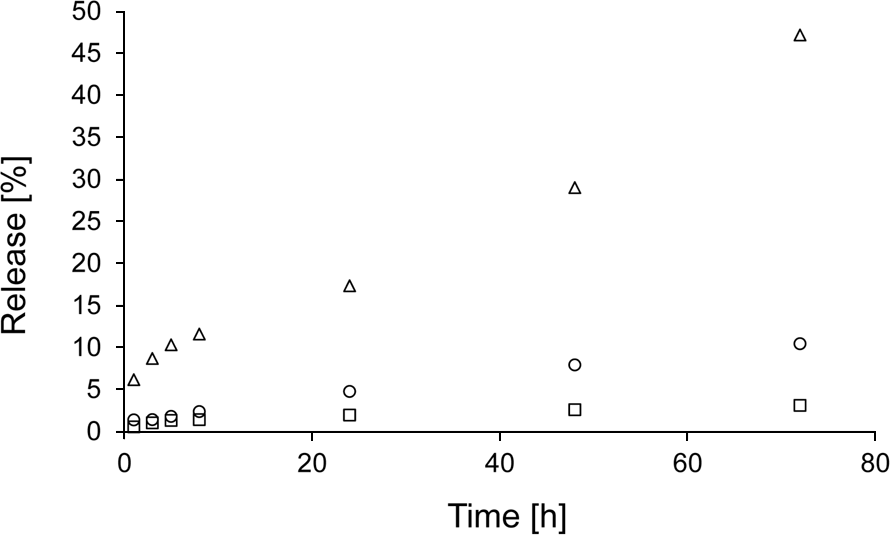
Release of PEG 2 from nanofibers prepared from PCL (open circles), PLA (open squares) and PVA (open triangles).

**Figure 6:**
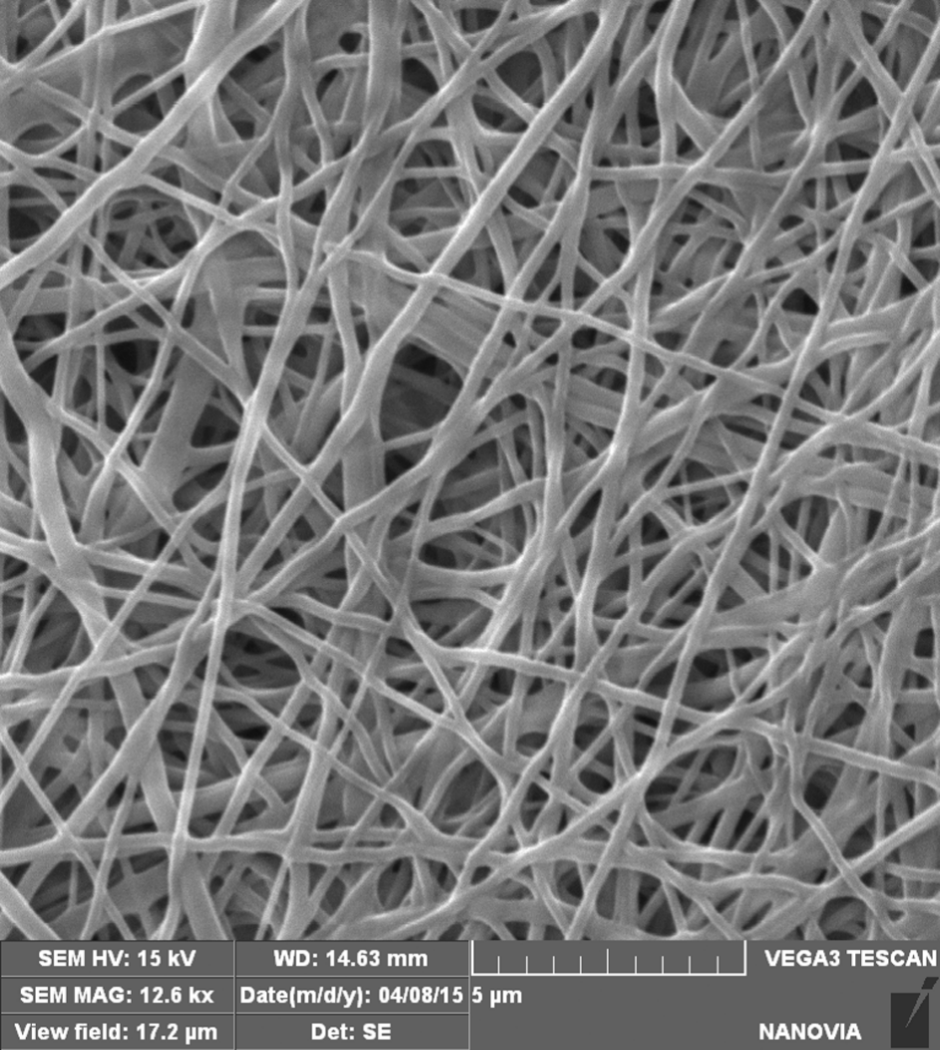
SEM image of PVA nanofibers containing PEG 20 after immersion into the water for 72 h.

The release of PEG molecules from PCL, PVA and PLA nanofibers varied, as evidenced by the amount of released model molecules after 72 h (Figure 4) compared to the amount of PEG 2 released from various nanofibers (Figure 5). PEGs of other molecular weights also followed similar trends. This effect may be partly due to the varied chemical composition of nanofibers as well as differences in their morphologies. From a chemical point of view, the fact that PEGs released fastest from PVA nanofibers can be explained by the interaction of PVA with water molecules during immersion – the PVA molecular structure contains hydroxy groups, which interact with water molecules at the expense of the interaction with PEG molecules, which are consequently released faster. The lower polarity of PCL and PLA and therefore weaker interaction with water molecules may result in such distinct differences between the release rates from PVA. The higher amount of released PEG from PVA brings the question whether it is not related to the dissolution of the fibers in an aqueous environment. Therefore, the SEM images of PVA nanofibers after release experiments were made (Figure 6). No significant changes in structure compared to starting material are apparent suggesting that the thermal crosslinking of the PVA fibers during the preparation ensures sufficient stability of the fibrous structure during the immersion into the water. Therefore, it can be concluded that the release kinetics are given by effects discussed above.

Despite attempts to ensure the similar nanofibrous structures, the varied morphology may be also responsible for the variation in the PEG release rate. The release of model molecules directly correlated with the specific surface areas and inversely correlated with the fiber diameters. A higher specific surface area provides a larger area for interaction with the surrounding liquid and consequent faster release of drug molecules. A larger fiber diameter increases the distance that molecules located in the middle of fiber must diffuse through to reach the periphery of the material, which prolongs release times. The porosity of nanofibers did not appear to affect the release rate. A larger porosity may increase the volume of liquid that surrounds the fibers and consequently accelerate the release, but this effect was not observed and may have been suppressed by other factors, i.e., the degree of hydrophilicity of nanofibers.

The obtained results showed the successful preparation of morphologically comparable nanofibrous materials with incorporated PEG molecules. It was demonstrated that combination of chemically different polymers and PEG of various molecular weights leads to materials with significantly different release kinetics of PEGs. These basic findings on relationships between PEG size and polymer structure on release kinetics were done in respect that even PEG serves as additive compound it has main effect on the release of potentially incorporated drug [27–28]. Especially, the addition of PEG has a great impact in systems with hydrophobic drugs (e.g., cyclosporine, paclitaxel) in which it can overcome the complication with homogenous dispersion of drug in the polymer matrix and mainly can facilitate the release into aqueous media in which the solubility of such drugs are very low.

## Conclusion

The release kinetics of polyethylene glycol molecules of various molecular weights from nanofibers prepared from polycaprolactone, polylactide and polyvinyl alcohol were assessed. The release rate and the total released amount positively correlated with molecular weight of the incorporated molecules. This trend was observed for all of the prepared nanofibrous carriers. The strongest effect appeared for PVA fibers containing hydroxy functional groups, i.e., 90% of PEG 20 model molecules released within 72 h. These findings can be applied to develop nanofibrous drug carriers for the local delivery of hydrophobic pharmacologically active compounds, because the release of auxiliary hydrophilic molecules can effectively control the drug release kinetics.

## Experimental

### Reagents

PCL (*M*_w_ ≈ 80 kDa) and trifluoroacetic acid were obtained from Sigma, St. Louis, MO, USA. PVA was provided by Nippon Gohsei, Osaka, Japan. PLA (*M*_w_ ≈ 100 kDa) was kindly provided as a sample from Natureworks, Blair, NE, USA. Phosphoric acid (85 wt % aqueous solution), *N*,*N*-dimethylformamide, tetrahydrofuran and dichloromethane were obtained from Penta, Prague, Czech Republic. The deionized water was produced by Milli-Q Millipore, Bedford, MA. Polypropylene nonwoven spunbond material used as a substrate for all polymers was purchased from ATEX, Milan, Italy. Polyethylene glycols with molecular weights of 2,000, 6,000, 10,000 and 20,000 were supplied by RAPP Polymere GmbH, Tübingen, Germany.

### Synthesis of derivatized polyethylene glycols

Prior to the derivatization reaction, the PEGs were dried as follows: the PEGs were dissolved in dry butyl acetate to obtain 10 wt % solutions, 4 Å molecular sieves were added and the solutions were stirred at room temperature (RT) for 3 days. The water content was determined using the Karl-Fischer method and was reduced from ca. 6000 ppm (not dried) to ca. 400 ppm (dried).

The derivatization reaction scheme is shown in Figure 1. Ten grams of PEG was dissolved in 80 mL of dry dioxane, and a 5% excess of the derivatization agent phenyl isocyanate was added. Dibutyltin dilaurate (500 ppm) was used to increase the reaction rate between phenyl isocyanate and the hydroxy groups and to ensure the double derivatization of PEGs. The reaction solution was stirred for 7 h. The reaction yield was 89.5%.

### Preparation of nanofibers

The nanofibers were prepared with the Nanospider^TM^ technology [21–22]. The process parameters used for each polymer were optimized in order to produce nanofiber samples of similar structures and a thickness (weight per unit area) of 5 g/m^2^. Model PEGs of various molecular weights were added to the polymer mixtures at a concentration of 3 wt %. PCL was dissolved in a 1:1 (w/w) mixture of tetrahydrofuran and *N*,*N*-dimethylformamide at a concentration of 10 wt %. The electrospinning parameters were 6 rpm, 15 cm and 5.5 kV/cm. The maximum temperature was 30 °C, and the maximum relative humidity was 35%. PLA was dissolved in a 2:1 (w/w) mixture of dichlormethane and trifluoroacetic acid at a concentration of 12 wt %. This mixture was held at 22 °C to maintain the viscosity of this polymer (due to very fast solvent evaporation). The electrospinning parameters were 4 rpm, 15 cm and 5.3 kV/cm. PVA was dissolved in water/phosphoric acid at a concentration of 11 wt %. The electrospinning parameters were 2 rpm, 13 cm and 45–55 kV/cm. The relative humidity was 25–30%, and the temperature was 22 °C. The PVA layers were crosslinked thermally in a drying oven at 145 °C for 15 min to reach their proper stability [27–28].

### Characterization of nanofibers

The structures of the prepared nanofibers were observed with a scanning electron microscope TS 5130 VEGA, TESCAN, Czech Republic. The samples were dried at 80 °C under vacuum overnight and platinum sputtered. Similar preparation procedure was used for the samples after immersion into the water for 72 h. The mean fiber diameter was determined from 30 measurements on the SEM images at a magnification of 5000×. Mercury porosimetry measurements were made using an Autopore IV 9500 porosimeter, Micromeritics, USA, and the specific surface areas were calculated based on nitrogen absorption/desorption isotherms recorded on an ASAP 2020 apparatus Micromeritics, USA.

### In vitro polyethylene glycol release assay

Samples of nanofibers (0.3 g) were placed into glass vials filled with 5 mL of distilled water. The samples were continuously shaken at room temperature for 5 days. At specific time points, 0.5 mL of solution was withdrawn, and the same amount of fresh water was added. The concentration of PEG was determined based on a calibration curve using a high performance liquid chromatography apparatus Shimadzu Prominence 20, USA with UV detection at a wavelength of 234 nm. The accumulative weight and relative percentage of the released PEGs were then calculated. This experiment was conducted for each of the above-mentioned nanofibers (PCL, PLA and PVA) and PEGs of various molecular weights.

## References

[1] Langer R, Peppas N A (2003). AIChE J.

[2] Geipel A, Goldschmidtboeing F, Jantscheff P, Esser N, Massing U, Woias P (2008). Biomed Microdevices.

[3] Nagy Zs K, Nyúl K, Wagner I, Molnár K, Marosi Gy (2010). eXPRESS Polym Lett.

[4] Ahmed I, Ponery A S, Nur-E-Kamal A, Kamal J, Meshel A S, Sheetz M P, Schindler M, Meiners S (2007). Mol Cell Biochem.

[5] Shih Y-R V, Chen C-N, Tsai S-W, Wang Y J, Lee O K (2006). Stem Cells.

[6] Barnes C P, Sell S A, Boland E D, Simpson D G, Bowlin G L (2007). Adv Drug Delivery Rev.

[7] Rieger K A, Birch N P, Schiffman J D (2013). J Mater Chem B.

[8] Leung V, Ko F (2011). Polym Adv Technol.

[9] Jayaraman K, Kotaki M, Zhang Y, Mo X, Ramakrishna S (2004). J Nanosci Nanotechnol.

[10] Huang Z-M, Zhang Y-Z, Kotaki M, Ramakrishna S (2003). Compos Sci Technol.

[11] Luo C J, Stoyanov S D, Stride E, Pelan E, Edirisinghe M (2012). Chem Soc Rev.

[12] Bellan L M, Craighead H G (2011). Polym Adv Technol.

[13] Sun B, Long Y Z, Zhang H D, Li M M, Duvail J L, Jiang X Y, Yin H L (2014). Prog Polym Sci.

[14] Bhardwaj N, Kundu S C (2010). Biotechnol Adv.

[15] Thompson C J, Chase G G, Yarin A L, Reneker D H (2007). Polymer.

[16] Hu X, Liu S, Zhou G, Huang Y, Xie Z, Jing X (2014). J Controlled Release.

[17] Persano L, Camposeo A, Tekmen C, Pisignano D (2013). Macromol Mater Eng.

[18] Jirsak O, Sanetrnik F, Lukas D, Kotek L, Martinova L, Chaloupek J (2006). Method of nanofibers production from polymer solution using electrostatic spinning and a device for carrying out the method. U.S. Patent.

[19] Lukas D, Sarkar A, Pokorny P (2008). J Appl Phys.

[20] Ryu Y J, Kim H Y, Lee K H, Park H C, Lee D R (2003). Eur Polym J.

[21] Xu X, Chen X, Ma P, Wang X, Jing X (2008). Eur J Pharm Biopharm.

[22] Jiang H, Wang L, Zhu K (2014). J Controlled Release.

[23] Son Y J, Kim W J, Yoo H S (2014). Arch Pharmacal Res.

[24] Saraf A, Baggett L S, Raphael R M, Kasper F K, Mikos A G (2010). J Controlled Release.

[25] Steele T W J, Huang C L, Widjaja E, Boey F Y C, Loo J S C, Venkatraman S S (2011). Acta Biomater.

[26] Herrmann S, Winter G, Mohl S, Siepmann F, Siepmann J (2007). J Controlled Release.

[27] Sirc J, Kubinova S, Hobzova R, Stranska D, Kozlík P, Bosakova Z, Marekova D, Holan V, Sykova E, Michalek J (2012). Int J Nanomed.

[28] Širc J, Hobzová R, Kostina N, Munzarová M, Juklíčková M, Lhotka M, Kubinová Š, Zajícová A, Michálek J (2012). J Nanomater.

